# An end-to-end framework for data lineage analysis covering link pattern recognition, fault diagnosis, and early warning

**DOI:** 10.1038/s41598-025-34522-1

**Published:** 2026-01-07

**Authors:** Rongxu Hou, Shaobo Zhang, Hongjiang Wang, Siwei Li, Yiying Zhang

**Affiliations:** 1 School of Computer Science and Technology, Shenyang Institute of Engineering, Shenyang, 110136 China; 2 China Gridcom Co., Ltd, Shenzhen, 518109 China; 3 Beijing Fibrlink Communications Co., Ltd., Beijing, 100071 China; 4 College of Artificial Intelligence, Tianjin University of Science and Technology, Tianjin, 300457 China

**Keywords:** Data lineage, Pattern recognition, Fault diagnosis, Engineering, Mathematics and computing

## Abstract

With the increasing complexity of data platforms, achieving real-time prediction and tracing of data link failures has become a critical issue that needs to be addressed. We proposes an End-to-End Full-Link intelligent analysis framework (EEFL) based on data lineage. This framework combines graph structures with deep learning algorithms to achieve link pattern recognition and fault warning. First, a dynamic data lineage graph model is constructed and topological features are extracted using a graph neural network (GNN). Through temporal edge weight optimization and semi-supervised clustering, typical link patterns are automatically classified. Second, a hybrid fault diagnosis model is designed, using a temporal convolutional network (TCN) to capture long-term dependencies between link metrics and combining it with a GNN to analyze topological mutations. This model accurately classifies various fault types, including data outages, latency anomalies, and data contamination. Finally, a dynamic threshold warning mechanism is introduced, combining Bayesian optimization and online learning to adaptively adjust alarm triggering conditions and effectively reduce false alarm rates. We verifies the generalization ability of the model using actual enterprise data and simulation data. Experimental results show that EEFL can achieve an average Acc of 92.73% across two datasets, which is significantly better than traditional methods and provides intelligent decision for data governance.

## Introduction

With the increasing integration of big data and intelligent technologies across various industries, including energy, finance, manufacturing, and smart cities, data centers have become vital hubs for storing and processing data sources while supporting upper-level business applications^1–3^. However, due to the exponential growth of data scale, the data flow between the middle platform and the business system usually shows the characteristics of complex links, frequent dynamics, and multi-source heterogeneity^4–6^. This not only significantly increases the difficulty of link management and pattern recognition, but also directly leads to a decline in business system performance and even service failures once a link interruption or delay occurs^7–9^. Therefore, it is urgent to build a data intelligent analysis and fault diagnosis method for the entire link to achieve data transmission pattern recognition, potential fault prediction, and efficient early warning management^10–12^.

Most traditional data link management methods rely on static modeling and manual rule-based configurations, which struggle to cope with the dynamic and evolving nature of data center environments^13–15^. Static methods fail to capture the complexities inherent in continuously changing data flows and dependencies. Meanwhile, data lineage technology, which tracks the flow and dependencies between data entities, has emerged as a critical tool for understanding data relationships and link modeling^16,17^. Although data lineage can offer insights into the data’s origin and transformation, automatically extracting topological patterns from large-scale dynamic data lineage graphs and recognizing link patterns, particularly when combined with runtime features, remains an unresolved challenge. To address this, recent advancements in graph neural networks (GNNs) have provided a new approach to learning from graph structures, offering powerful capabilities for topological pattern extraction^18–20^. In dynamic and complex link environments, where failures such as link interruptions, latency anomalies, and data corruption are common, traditional fault detection methods, which typically rely on statistical models or shallow machine learning, fall short in simultaneously considering the temporal characteristics and topological dependencies of the data flow. While temporal convolutional networks (TCNs) have excelled in capturing long-term dependencies and detecting sequence anomalies^21,22^, they lack the ability to incorporate graph structures. Conversely, methods based solely on GNNs can model topological dependencies effectively but are inadequate in capturing temporal dynamics. Thus, combining TCNs and GNNs into a hybrid model represents a crucial breakthrough for accurate classification and diagnosis of various types of link faults^23–25^.

In addition to accurate fault classification, the rationality of the early warning mechanism is directly related to the reliability of system operations and maintenance^26,27^. Traditional fixed threshold strategies often suffer from high false alarm rates or high false negative rates, and are difficult to adapt to the dynamic characteristics of data distribution over time^28,29^. In recent years, Bayesian optimization and online learning methods have shown potential in threshold adaptive adjustment^30,31^. In particular, the follow-the-regularized-leader (FTRL) algorithm^32,33^, due to its online update capabilities and convergence stability, can effectively reduce false alarm and false negative rates. Therefore, introducing a dynamic threshold mechanism and combining it with an online learning strategy is of great significance for improving the intelligence level of early warning systems.

Although existing methods have conducted in-depth research on data link fault diagnosis, they are difficult to meet the operation and maintenance requirements of complex data middle platforms. This is mainly due to the following problems: (1) Static lineage modeling cannot adapt to the dynamic nature of the link. (2) Single feature modeling cannot take into account both timing and topological correlations. (3) Existing hybrid models often lack a comprehensive design for feature fusion, which is crucial for leveraging the complementary strengths of temporal and topological learning. To address the above problems, this paper proposes an end-to-end full-link intelligent analysis and diagnosis method (EEFL) based on data lineage. The main contributions of this paper are as follows:

1. A data-driven link pattern recognition method is proposed. Through the joint learning of dynamic lineage graph modeling and weighted GNN, the automatic recognition of complex link structures is achieved, breaking through the limitations of traditional manual rules and static modeling.
2. A TCN-GNN fusion diagnostic model is constructed. TCN and GNN are combined to capture the dynamic and structural characteristics of link runtime, and link fault diagnosis is achieved by leveraging time series and topological dependencies.
3. A dynamic threshold warning mechanism is introduced. Bayesian optimization and FTRL online learning are combined to propose an adaptive threshold adjustment method to effectively cope with dynamic changes in data distribution and significantly reduce the false alarm rate and missed alarm rate of the early warning system.

The subsequent chapters of this paper are organized as follows. “Relevant basic theories” section presents related work and theoretical foundations, systematically expounding the core theories of GNNs and TCNs. “An end-to-end full-link intelligent analysis framework based on data lineage” section provides a detailed design of the EEFL framework, sequentially elaborating on the implementation logic and mathematical derivation of the dynamic graph-based link pattern recognition module, the TCN-GNN hybrid fault diagnosis module, and the Bayesian-FTRL dynamic threshold warning module. “Experimental analysis” section presents experimental design and results analysis, quantitatively analyzing the performance advantages of EEFL in link pattern recognition, fault diagnosis, and warning by comparing it with mainstream methods. “Conclusion” section summarizes the contributions of this paper and future research plans.

## Relevant basic theories

### Graph neural network

Graph neural network (GNN)^34^ is a type of deep learning model specifically designed to process non-Euclidean structured data. Their core advantage lies in their ability to model complex topological dependencies through information interaction between nodes. This feature makes them naturally adaptable to data lineage link analysis. Data lineage can essentially be abstracted as a graph structure consisting of “data entities (nodes) - dependency relationships (edges)”, and GNNs can effectively capture the implicit association features in such structures, providing technical support for link pattern recognition and fault tracing.

A graph can be represented as $G = (V,E)$, where *V* is the set of nodes, *E* is the set of edges. Each node corresponds to a data entity in the data center, such as a database table, ETL job, API interface, etc. In order to quantitatively describe the connection relationship between nodes, the adjacency matrix *A* is introduced as Eq.1.

1 $$\begin{aligned} A_{ij} = {\left\{ \begin{array}{ll} 1, & \text {if } (v_i, v_j) \in E \\ 0, & \text {else} \end{array}\right. } \end{aligned}$$

The adjacency matrix satisfies the symmetry, which can effectively simplify the feature propagation calculation in the subsequent graph convolution process. In order to characterize the connection strength of the nodes, the degree matrix *D* is designed as $D_{ii} = \sum _{j=1}^{N} A_{ij}$. Where *A* is the adjacency matrix. The degree matrix can not only reflect the association of nodes in the bloodline link, but also be used for the normalization of the subsequent adjacency matrix to avoid feature offset caused by node degree differences during graph convolution.

The core idea of GNN is that nodes update their own representations by exchanging information with their neighbors, and iterate continuously until convergence or reaching a set number of layers. At the *l*th layer, the representation vector of each node *v* is $h_v^{(l)}$, and the next layer representation $h_v^{(l + 1)}$ is obtained by aggregating the features of neighboring nodes. The calculation process is shown in Eqs. 2 and 3.

2 $$\begin{aligned} \textbf{m}_v^{(l+1)}= & \textrm{AGGREGATE}^{(l)} \left( \left\{ {h}_u^{(l)}, \; \forall u \in \mathcal {N}(v) \right\} \right) \end{aligned}$$

3 $$\begin{aligned} {h}_v^{(l+1)}= & \sigma \left( W^{(l)} \cdot \textrm{COMBINE} \left( {h}_v^{(l)}, {m}_v^{(l+1)} \right) \right) \end{aligned}$$

where ${AGGREGATE}^{(l)}$ is the neighbor feature aggregation function of the *l*-th layer, ${m}_v^{(l+1)}$ is the aggregated information from the neighbors, *COMBINE* is the combination of the node’s own features and neighbor information, $W^{(l)}$ is the trainable weight matrix of the *l*th layer, and $\sigma ()$ is the activation function.

It is worth noting that in the practical application of data lineage link analysis, the original GNN model requires two optimizations. First, to address the dynamic nature of lineage links, the static adjacency matrix is expanded to a dynamic one that changes over time. This allows the model to capture the temporal evolution of link topology. Second, to account for the data dependencies between edges within lineage links, the binary adjacency matrix is expanded to a weighted adjacency matrix. Edge weights are adjusted to focus on key links. These optimizations enable GNNs to more accurately adapt to the characteristics of data lineage scenarios, laying the foundation for high-precision link pattern recognition.

### Temporal convolutional network

TCN^35^ is a deep learning architecture designed specifically for processing time series data, which demonstrated significant advantages in various sequence modeling tasks in recent years. In particular, it has unique value in capturing the dynamic patterns and long-term dependencies of indicators in data links over time. In data link analysis scenarios, the various indicators generated by the data middleware essentially constitute a set of sequence data with strong time series characteristics. Its values at different times not only reflect the current operating status of the link, but also contain historical information from previous times. This time dependency is crucial for accurately diagnosing link faults and predicting future status.

TCNs often use a residual structure to ensure the trainability of deep networks. A typical residual block consists of two or more layers of causal dilated convolution, activation, normalization, dropout, and then a residual connection. For the input sequence $x = ({x_1},{x_2}, \ldots ,{x_T})$, the output ${y_T}$ of the residual block is calculated in Eqs. 4, 5 and 6.

4 $$\begin{aligned} & z^{(1)}_t = \sigma \big ( \textrm{Dropout}(\, \textrm{Conv}^{(1)}_{\text {causal},d^{(1)}}(x) \,) \big ) \end{aligned}$$

5 $$\begin{aligned} & z^{(2)}_t = \sigma \big ( \textrm{Dropout}(\, \textrm{Conv}^{(2)}_{\text {causal},d^{(2)}}(z^{(1)}) \,) \big ) \end{aligned}$$

6 $$\begin{aligned} & y_t \;=\; \textrm{LayerNorm}\big ( x_t + W_{res} *x_t + z^{(2)}_t \big ) \end{aligned}$$

where $\sigma ()$ is the activation function, $\mathrm{{D}}ropout()$ is random dropout operation, *Conv*() is causal convolution, ${W_{res}}$ is convolution matrix, and $W_{res}$ is convolution matrix. And the causal one-dimensional convolution is designed as Eq. 7.

7 $$\begin{aligned} (y *_{\text {causal}} f)_t \;=\; \sum _{i=0}^{k-1} f_i \, x_{t-i} \qquad \text {(where } x_{s}=0\text { when } s\le 0\text {)} \end{aligned}$$

where ${(y{*_{\mathrm{{causal}}}}f)_t}$ represents the causal convolution result at time *t*, *k* is the length of the convolution kernel, $f_i$ represents the *i*th parameter of the convolution kernel, $x_{t-i}$ represents the value of the input sequence *x* at trial $t-i$.

However, simple causal convolution has limitations when capturing long-range temporal dependencies. As the dependency distance increases, the number of convolution kernel layers required increases linearly, leading to a sharp increase in computational effort and model complexity. When constructing TCN models, a Temporal Block structure is typically formed by stacking multiple layers of causal convolution and dilated convolution as the basic building block of the network. The computational process is shown in Eq. 8.

8 $$\begin{aligned} h^{(l)}_{t,j} \;=\; \sum _{c=1}^{C_{in}} \sum _{i=0}^{k-1} W^{(l)}_{j,c,i}\; h^{(l-1)}_{t-i,c} \;+\; b^{(l)}_j \end{aligned}$$

Dilated convolution inserts gaps between convolution kernel elements to exponentially expand the receptive field (RF) of the convolution kernel, effectively capturing long-distance temporal dependencies without significantly increasing the number of parameters and computational complexity. The dilated convolution is shown in Eqs. 9.

9 $$\begin{aligned} \textrm{RF} \;=\; 1 \;+\; \sum _{l=0}^{L-1} (k-1)\, d^{(l)} \end{aligned}$$

where *k* is the convolution kernel length, *d* is the dilation rate, *l* is the layer index, $h_t^l$ is the hidden representation of the *l*th layer at time *t*, and $*$ is a one-dimensional discrete convolution.

### Follow-the-regularized-leader

FTRL is a state-of-the-art online optimization algorithm designed for sequential decision-making problems, particularly in dynamic or non-stationary environments where data distributions may shift over time. It has demonstrated exceptional performance in large-scale, high-dimensional applications, due to its ability to produce sparse, stable, and highly accurate models.

The core principle of FTRL is to make a decision at each time step *t* by selecting a parameter vector $\theta _t$ that minimizes a function combining historical performance with a regularization penalty. This strategy elegantly balances two objectives: exploiting past information and preventing overfitting or instability.

Let $L_i(\theta )$ be the loss function at a past time step *i*, and let $g_i = \Delta L_i(\theta _i)$ be its gradient. The FTRL algorithm selects the next parameter $\theta _{t+1}$ by solving the following optimization problem:

10 $$\begin{aligned} {\theta _{t + 1}} = \mathop {\arg \min }\limits _\theta \left\{ {\sum \limits _{i = 1}^t {{g_i}\theta + R(\theta )} } \right\} \end{aligned}$$

where ${\sum \limits _{i = 1}^t {{g_i}\theta } }$ encourages the selection of a parameter $\theta$ that would have performed best and $R(\theta )$ is a regularization function that penalizes model complexity to ensure stability and prevent drastic changes in the parameters.

## An end-to-end full-link intelligent analysis framework based on data lineage

**Fig. 1:**
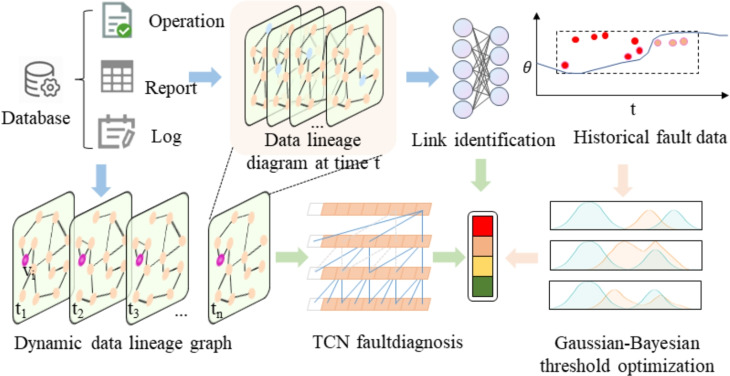
End-to-end full-link intelligent analysis framework based on data lineage.

As shown in the Fig. 1, this is an end-to-end full-link intelligent analysis framework based on data lineage. Its main ideas are: first, build a dynamic data lineage graph and introduce GNN to realize the automatic extraction of topological dependencies between data entities; then optimize edge weights based on time series features to achieve high-precision recognition of link patterns such as linear links, star topologies, and ring dependencies; then, use TCN to capture the long-term dependencies of link operation indicators, and combine GNN to analyze topological changes to achieve accurate classification of multiple types of faults; finally, design a dynamic threshold warning mechanism, combine Bayesian optimization and FTRL algorithms to achieve adaptive adjustment and continuous optimization of thresholds, effectively deal with data distribution drift, and significantly reduce false positives and missed reports.

### Link pattern recognition based on dynamic graph structure

**Fig. 2:**
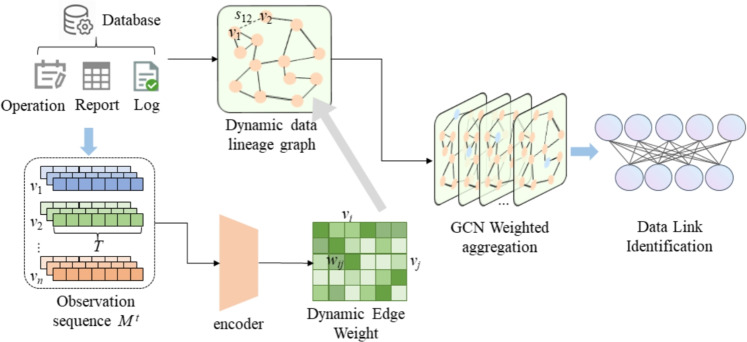
Link pattern recognition architecture based on dynamic graph structure.

Existing research on data link pattern recognition relies heavily on static lineage modeling, which is unable to capture the real-time evolution of link topology and quantify the differences in business importance of different dependencies. This results in insufficient pattern recognition accuracy in complex scenarios and is unable to support the refined operation and maintenance requirements of the data center. To this end, this paper designs a link pattern recognition module based on a dynamic data lineage graph, as shown in Fig. 2.

First, we overcome the limitations of static graphs, which lack a time dimension and are binary in edge weights, and construct a timestamp-based quadruple model to dynamically characterize link topology and dependency strength. Second, we propose a weighted adjacency matrix that integrates time series statistical features with business attributes to address the shortcomings of traditional models, which prioritize structure over semantics. Finally, we optimize the feature propagation mechanism based on a weighted graph convolutional neural network (WGCN) and use a normalization strategy to alleviate the gradient bias caused by uneven edge weights, thereby improving the recognition accuracy of complex patterns. The specific process is as follows.

In order to describe the structural relationship between data, we first build a dynamic data lineage graph ${G^t} = (V,{E^t},{A^t},{X^t})$, which aggregates the original events/logs and static metadata into a weighted graph that evolves over time. Specifically, it includes core entities such as node type, CPU usage, memory utilization, and historical fault frequency. The edge set $E^t$ is a dynamic edge set that evolves over time, reflecting the real-time dependency between nodes. The adjacency matrix $A^t$ is different from a static binary matrix and can quantify the dependency strength and temporal dynamics of the edges.

Raw events, logs, and static metadata are aggregated into a weighted graph that evolves over time. The original observations of edge (*i*, *j*) in the sliding window $[t - T + 1, \ldots ,t]$ are denoted as Eq. 11

11 $$\begin{aligned} M_{ij}^t = \big [\, m_{ij}^{t-T+1}, \; m_{ij}^{t-T+2},\; \dots ,\; m_{ij}^{t}\,\big ] \in \mathbb {R}^{T\times F_e} \end{aligned}$$

where *V* is a fixed set of nodes, *t* is a discrete time index, $M_{ij}^t$ is the raw time observation of edge (*i*, *j*) in the time window. Its mean and variance are shown in Eq. 12

12 $$\begin{aligned} \bar{m}_{ij}^t = \frac{1}{T}\sum _{s=t-T+1}^{t} m_{ij}^s,\quad \textrm{Var}_{ij}^t = \frac{1}{T}\sum _{s=t-T+1}^{t} (m_{ij}^s - \bar{m}_{ij}^t)^2 \end{aligned}$$

where $\bar{m}_{ij}^t$ reflects the average running state of edge (i,j) in the window, and ${Var}_{ij}^t$ reflects the degree of state fluctuation.

Then, a binary initial edge set is constructed based on the *log*, and edges are considered to exist when dependencies have occurred or when threshold filtering is used. The process is shown in Eq. 13.

13 $$\begin{aligned} {E^t} = \{ (i,j)\mid \mathrm{{count}}_{\mathrm{{events,ij}}}^{t - T + 1:t} > \tau \} \end{aligned}$$

where $E^t$ is a set at time *t*, $\mathrm{{count}}_{\mathrm{{events,ij}}}^{t - T + 1:t}$ indicates the number of events that occur on edge (i,j) within the sliding window, $\tau$ is the event frequency threshold. The initial (unnormalized) adjacency matrix is calculated in Eq. 14.

14 $$\begin{aligned} A^t_{ij,0} = {\left\{ \begin{array}{ll} s_{ij}^t, & (i,j)\in E^t\\ 0, & \text {otherwise} \end{array}\right. } \end{aligned}$$

where $A^t$ is the weighted adjacency matrix at time *t*, The time series observation matrix ${M}_{ij}^t$ of the edge is encoded into a fixed-length vector $e_{ij}^t$ through TCN. The process is shown in Eqs. 15 and 16.

15 $$\begin{aligned} H^{(l)}_{s, j} \;=\; \sigma \!\left( \sum _{c=1}^{C_{in}} \sum _{q=0}^{k-1} W^{(l)}_{j,c,q} \; H^{(l-1)}_{s - d^{(l)} q,\; c} \;+\; b^{(l)}_j\right) \end{aligned}$$

16 $$\begin{aligned} e_{ij}^t = \textrm{Linear}\big ( \textrm{Pool}\big ( H^{(L)} \big ) \big ) \in \mathbb {R}^{d_e} \end{aligned}$$

In Eq. 16, $H^{(l)}$ is the node representation at the *l*-th layer at time *t*, $e_{ij}^t$ is the edge embedding of edge (*i*, *j*) at time *t*.

Based on this, a learnable mapping is used to map the edge embeddings $e_{ij}^t$ and optional static meta data $s_{ij}$ to scalar weights $\omega _{ij}^t$. As shown in Eq. 17.

17 $$\begin{aligned} \tilde{w}_{ij}^t = \mathrm{{MLP}}([{\hspace{0.55542pt}} e_{ij}^t{\hspace{0.55542pt}} {\hspace{0.55542pt}} {s_{ij}}{\hspace{0.55542pt}} ]),\qquad w_{ij}^t = \sigma (\tilde{w}_{ij}^t) \in (0,1) \end{aligned}$$

where $w_{ij}^t$ is the final weight of edge (*i*, *j*) at time *t*. Then, form the final weighted adjacency matrix as shown in Eq. 18.

18 $$\begin{aligned} A^t_{ij} = {\left\{ \begin{array}{ll} w_{ij}^t, & (i,j)\in E^t,\\ 0, & \text {otherwise} \end{array}\right. } \end{aligned}$$

Then self-loops are introduced and symmetric normalized for GCN propagation. As shown in Eq. 19

19 $$\begin{aligned} \tilde{A}^t = A^t + I_N,\qquad \tilde{D}^t_{ii} = \sum _{j} \tilde{A}^t_{ij},\qquad \hat{A}^t = (\tilde{D}^t)^{-\frac{1}{2}} \tilde{A}^t (\tilde{D}^t)^{-\frac{1}{2}} \end{aligned}$$

where $\tilde{A}^t$ is the weighted adjacency matrix, $I_N$ is the identity matrix and $\tilde{D}^t_{ii}$ is the degree matrix. Based on the weighted GCN, the node representation after *L* layers is calculated as Eq. 20.

20 $$\begin{aligned} H^{(l+1),t} = \sigma \big ( \hat{A}^t \, H^{(l),t} \, W^{(l)} \big ), \qquad H^{(0),t} = X^t \end{aligned}$$

Since $\hat{A}^t$ already contains the dynamic weight $w_{ij}^t$ given by the temporal encoder, the GCN aggregation is automatically “driven by the optimized edge weights,” thereby realizing the influence of temporal features on topological dependencies. If a pattern classification is performed on an end-to-end link, the subgraph representation $z_G^t$ is first obtained from the node representation, and then the classification is performed as Eq. 21.

21 $$\begin{aligned} \beta _v^t = \frac{\exp \!\big ( q_p^\top \tanh ( W_p H_v^{(L),t} ) \big )}{\sum _{u\in V_G}\exp \!\big ( q_p^\top \tanh ( W_p H_u^{(L),t} ) \big )}, \quad z_G^t = \sum _{v\in V_G} \beta _v^t \, H_v^{(L),t} \end{aligned}$$

In Eq. 21, $z_G^t$ is the subgraph representation at time *t*, $\beta _v^t$ is the attention weight of node at time t, $q_p$ is a learnable query vector, $W_p$ is the weight matrix. The loss function ${L}_{\text {CE}}$ for model training is designed as Eq. 22.

22 $$\begin{aligned} {L}_{\text {CE}} = -\sum _{c=1}^{C} y_c^t \log \hat{y}_c^t \end{aligned}$$

After the above steps, the construction of a dynamic data lineage graph model and the automatic classification of typical link patterns can be achieved.

### Hybrid diagnostic model based on TCN-GNN

**Fig. 3:**
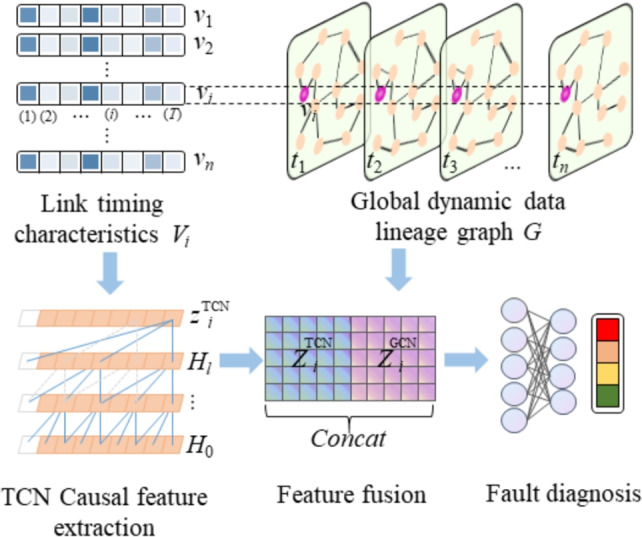
Architecture diagram of hybrid diagnostic model based on TCN-GNN.

Because single time series models lack the ability to model topological dependencies, single graph models struggle to capture long-term temporal correlations. This results in low classification accuracy for various fault types, such as data interruptions, latency anomalies, and data contamination. To address this, this paper proposes a hybrid diagnosis model based on TCN and GNN, as shown in Fig. 3. First, TCN is used to capture the long-term temporal dependencies of link metrics, while GCN leverages the data’s topological structure to mine for structural correlations. Then, through a feature interaction fusion mechanism, the synergy between the two features is enhanced, addressing the insufficient diagnostic accuracy of single models in complex link scenarios. The specific steps are as follows.

For the global graph $G = (V,E,W)$, the link timing features corresponding to each node ${v_i} \in V$ are shown in 23.

23 $$\begin{aligned} {X_i} = [x_i^{(1)},x_i^{(2)}, \ldots ,x_i^{(T)}] \in {R^{T \times F}} \end{aligned}$$

The *T* is the time step, *F* is the dimension of each step feature. For the time series data of each node, apply TCN multi-layer causal expansion convolution. The process is shown in Eq. 24.

24 $$\begin{aligned} H_i^{(l)}(t) = \sigma \left( \sum _{k=0}^{K-1} W^{(l)}(k) \cdot H_i^{(l-1)}(t - d \cdot k) + b^{(l)} \right) \end{aligned}$$

where *l* represents *l*th layer, $H_i^{(0)}(t) = x_i^{(t)} \in {R^F}$, $W^{(l)}$ is the convolution kernel weight matrix, *d* is the expansion factor, $\sigma$ is the activation function. After *L* layers of stacking, the temporal feature representation of each node is obtained as Eq. 25

25 $$\begin{aligned} z_i^{\text {TCN}} = \frac{1}{T} \sum _{t=1}^T H_i^{(L)}(t) \end{aligned}$$

Based on the data lineage graph weight matrix *W*, a symmetric normalized adjacency matrix is constructed as Eq. 26

26 $$\begin{aligned} \tilde{W} = D^{-\frac{1}{2}} (W + I) D^{-\frac{1}{2}}, \quad D_{ii} = \sum _j (W_{ij} + I_{ij}) \end{aligned}$$

*I* is the identity matrix. Define the node input feature matrix as Eq. 27

27 $$\begin{aligned} {Z^{TCN}} = \left[ \begin{array}{l} Z_1^{TCN}\\ Z_2^{TCN}\\ \vdots \\ Z_N^{TCN} \end{array} \right] \end{aligned}$$

That is, the time series features of each node are input into GCN as the initial node features. The propagation rule of the GCN layer is shown in Eq. 28

28 $$\begin{aligned} z_i^{GCN} = \sigma \left( {\tilde{A}H_{GCN}^{(l\mathrm{{ - 1}})}W_{GCN}^{^{(l)}}} \right) \end{aligned}$$

where $W_{GCN}^{^{(l)}}$ is the weight matrix, $H_{GCN}^{(l - 1)}$ is the depth feature after the temporal feature is propagated through the (l-1) layer and $\sigma$ is the activation function. After propagating through ${L'}$ layers, the final node embedding representation is obtained as ${Z^{GCN}} = {H^{(L')}}$. For node ${v_i}$, its GCN representation $Z^{GCN}$ and the corresponding time series feature $Z^{TCN}$ are fused as Eq. 29.

29 $$\begin{aligned} z_i = \textrm{ReLU} \left( W_f \begin{bmatrix} z_i^{\text {TCN}} \\ z_i^{\text {GCN}} \end{bmatrix} + b_f \right) \end{aligned}$$

In Eq. 29, $W_f$ is the weight matrix and $b_f$ is the bias. The fault class probability is then predicted using MLP, as shown in Eq. 30.

30 $$\begin{aligned} \hat{y}_i = \textrm{softmax} \big ( \textrm{MLP}(z_i) \big ) \end{aligned}$$

Taking all nodes in the entire graph as training samples, and the loss is defined as Eq. 31

31 $$\begin{aligned} {L} = - \sum _{i=1}^N \sum _{c=1}^C y_{i,c} \log \hat{y}_{i,c} \end{aligned}$$

where $y_{i,c}$ is the true label and $\hat{y}_{i,c}$ is the predicated label. Based on the above steps, accurate classification of multiple types of faults can be achieved.

### Design of dynamic threshold alarm mechanism

**Fig. 4:**
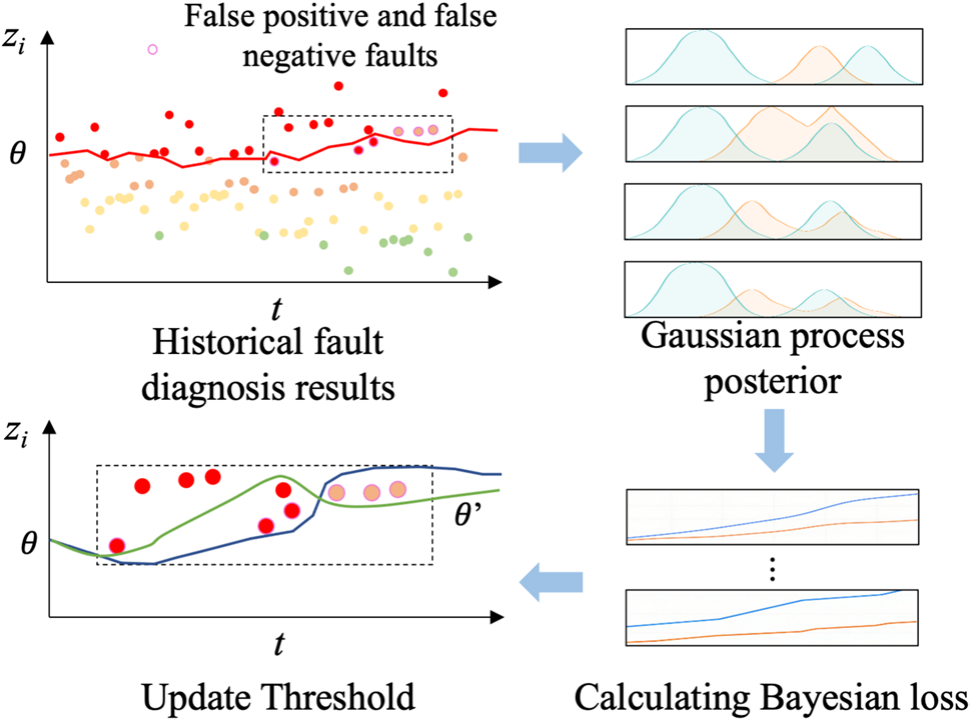
Design of dynamic threshold alarm mechanism.

To address the high false alarm and missed alarm rates caused by the inability of traditional fixed thresholds to adapt to dynamic drift in data distribution, this section designs a dynamic threshold alerting mechanism that combines Bayesian optimization and FTRL online learning, as shown in Fig. 4. This mechanism uses the fault probability output by the fault diagnosis model as input and implements a two-stage strategy of offline global optimization and online dynamic fine-tuning to achieve adaptive adjustment of the alert threshold. This ensures global optimality of the threshold while rapidly responding to real-time data changes. Ultimately, it significantly reduces the false alarm and missed alarm rates of the early warning system, providing accurate risk warnings for data center operations and maintenance. The specific process is as follows.

According to the results in the previous section, the fault probability output by the model is ${z_i} \in [0,1]$. The alarm threshold at the current time point *t* is ${\theta _t}$, when ${z_i} > {\theta _t}$. An alarm will be triggered. In order to find the optimal threshold to smooth the false alarm rate and the missed alarm rate, the objective function is designed as Eq. 32.

32 $$\begin{aligned} J(\theta ) = \lambda _1 \cdot \textrm{FPR}(\theta ) + \lambda _2 \cdot \textrm{FNR}(\theta ) \end{aligned}$$

where $\lambda _1$ and $\lambda _2$ are the weighting coefficients for false positives FPR and false negatives FNR. Because $J(\theta )$ is a black box function that cannot be explicitly solved, and directly traversing all possible $\theta$ would result in a surge in computational complexity, a Gaussian process (*GP*) surrogate model is established for $J(\theta )$ using historical data to efficiently approximate the true loss function. Through historical data, a *GP* agent model is established for $J(\theta )$, as shown in Eq. 33

33 $$\begin{aligned} J(\theta ) \sim {GP}(\mu (\theta ), k(\theta , \theta ')) \end{aligned}$$

The $\mu (\theta )$ uses the constant mean function $\mu _0$, which is the average loss of historical threshold samples. The kernel function $k(\theta , \theta ')$ uses the radial basis function (RBF) kernel, which can effectively fit the continuous change trend of $J(\theta )$ is robust to noise data, and is suitable for a small number of abnormal samples in historical data.

First, the GP posterior is continuously updated using the collected threshold-loss pair data, and then the next trial threshold is determined by the acquisition function (such as expected improvement), and finally iterates until convergence to obtain the optimal threshold.

Bayesian optimization is mainly aimed at batch updates. In the face of dynamic changes in data distribution, an online learning threshold adjustment strategy based on follow the regularized leader (FTRL) is designed. Let the threshold at step *t* be ${\theta _t}$ and the loss function (expressed as loss gradient) be ${g_t} = {\nabla _\theta }{l_t}({\theta _t})$. The iteration rule of FTRL is processed in Eq.34.

34 $$\begin{aligned} \theta _{t+1} = \arg \min _\theta \left\{ \sum _{i=1}^t g_i \theta + \frac{1}{2} \sum _{i=1}^t \sigma _i \theta ^2 + \lambda _3 |\theta | \right\} \end{aligned}$$

where $\sum _{i=1}^t g_i \theta$ is the cumulative historical loss gradient term, ensuring that the new threshold minimizes the sum of the losses of all past steps. $\frac{1}{2} \sum _{i=1}^t \sigma _i \theta ^2$ is the adaptive learning rate regularization term, and ${\sigma _i}$ is the learning rate adjustment parameter, which adaptively decreases with the number of iterations to avoid drastic fluctuations in the threshold in the later stages. The analytical solution can be expressed as Eq. 35.

35 $$\begin{aligned} \theta _{t+1} = - \frac{1}{\sum _{i=1}^t \sigma _i} \left( \sum _{i=1}^t g_i + \lambda _3 \cdot \textrm{sign}(\theta _{t+1}) \right) \end{aligned}$$

Combining the two comprehensive processes, in the initial stage, Bayesian optimization is used to batch find the initial threshold ${\theta _t}$. In real-time online data, FTRL is used to continuously fine-tune the threshold based on the loss gradient of new samples, as shown in Eq. 36.

36 $$\begin{aligned} \theta _t = \theta _{t-1} + \Delta \theta _t \end{aligned}$$

where $\Delta \theta _t$ is calculated by FTRL optimization. This mechanism adapts to data distribution drift, dynamically updates the threshold, and significantly reduces false positives and negatives. The schematic diagram of the overall process of the method is shown in the figure:

## Experimental analysis

### Actual enterprise data center evaluation

The verification dataset used in this section comes from the production link operation records of the actual enterprise data center, which includes two parts: topology data and link operation sequence data. Topology data primarily includes key data entities in the data center, such as database tables, ETL jobs, API interfaces, and data files. Link runtime data primarily includes latency, throughput, packet loss rate, and error rate. Specifically, containing 1,240 nodes (services, APIs, or databases) and 5,870 edges (data flows). Each node has 32 features (CPU load, I/O latency, memory utilization, throughput, etc.), and each edge has 8 temporal attributes (delay, packet loss rate, transfer volume, etc.). Labels are derived from operational logs and annotated as normal, data interruption, latency anomaly, or data pollution. 70% of the data is used for training and 30% of the data is used for testing. The evaluation indicators used in the experimental results are accuracy, recall, precision, and ${F_1}$score^36^, as shown in Eqs. 37, 38, 39 and 40.

37 $$\begin{aligned} & Accuracy = \frac{{TP + TN}}{{TP + FP + TN + FN}} \times 100\% \end{aligned}$$

38 $$\begin{aligned} & Precision\mathrm{{ = }}\frac{{TP}}{{TP + FP}} \times 100\% \end{aligned}$$

39 $$\begin{aligned} & Recall = \frac{{TP}}{{TP + FN}} \times 100\% \end{aligned}$$

40 $$\begin{aligned} & {F_1}score = \frac{{2 \times Precision \times Recall}}{{Precision + Recall}} \end{aligned}$$

Where *TP* and *TN* are the numbers of samples predicted correctly, and *FP* and *FN* are the numbers of samples predicted incorrectly.

**Fig. 5:**
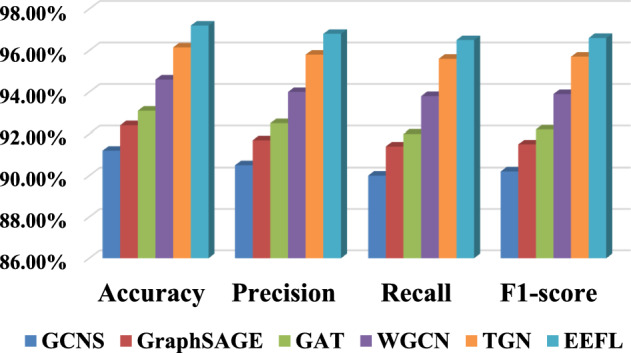
Comparison results of link pattern recognition of various algorithms.

1. Verification experiment of link pattern recognition: In order to verify the effectiveness of the proposed method EEFL in link identification, EEFL is compared with the basic GCN based on static adjacency matrix (GCNS)^37^, graph sampling and aggregate (GraphSAGE)^38^, graph attention network (GAT)^39^, weighted GCN (WGCN)^40^ based on temporal feature optimization of edge weights and temporal graph network (TGN). The results are shown in Table 1 and Fig. 5.

**Table 1:** Comparison results of link pattern recognition of various algorithms.

Algorithm	Accuracy	Precision	Recall	${F_1}$score
GCNS	91.20%±0.45%	90.50%±0.51%	90.00%±0.62%	90.20%±0.48%
GraphSAGE	92.40%±0.38%	91.70%±0.42%	91.40%±0.45%	91.50%±0.35%
GAT	93.10%±0.35%	92.50%±0.39%	92.00%±0.41%	92.20%±0.33%
WGCN	94.60%±0.29%	94.00%±0.33%	93.80%±0.35%	93.90%±0.28%
TGN	96.15%±0.25%	95.80%±0.28%	95.60%±0.31%	95.70%±0.24%
EEFL	97.20%±0.21%	96.80%±0.23%	96.50%±0.26%	96.60%±0.19%

In experimental validation of link pattern recognition, our approach demonstrated significant advantages over several mainstream graph neural network models. Specifically, while GCNS can extract basic topological features when processing link structures, its convolutional operator only considers the mean aggregation of neighboring nodes, making it difficult to effectively distinguish different types of link patterns, resulting in an accuracy of only 91.20%. GraphSAGE enhances representational capabilities through sampling and aggregation strategies, improving accuracy to 92.40%. However, it still struggles with discriminative power in complex topologies (such as cyclic dependencies). GAT utilizes an attention mechanism to assign different weights to neighbors, better capturing the dependencies of key nodes and improving accuracy to 93.10%. WGCN further incorporates link timing features to optimize edge weights, achieving improved performance to 94.60%. In contrast, the data lineage-based WGNN model proposed in this paper, after simultaneously considering topological dependency and temporal dynamic characteristics, can more accurately distinguish three types of patterns: linear chain, star topology, and cyclic dependency. It ultimately achieves the best performance in accuracy, recall, precision, and ${F_1}$score (accuracy 97.20%, recall 96.50%, precision 96.80%, ${F_1}$score 96.60%), verifying the effectiveness and robustness of the proposed method in full-link intelligent analysis.

**Fig. 6:**
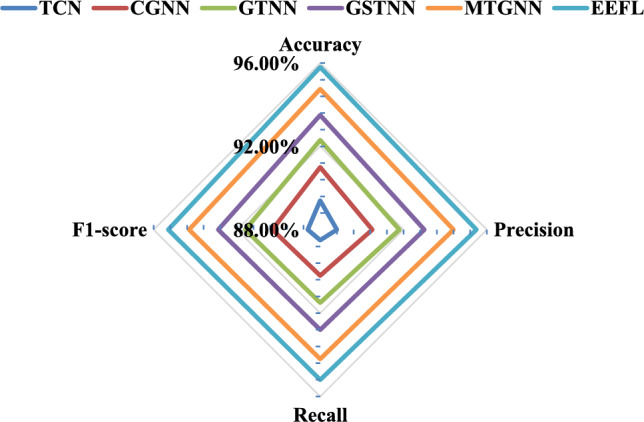
Comparison results of fault diagnosis.

(2) Verification experiment of fault diagnosis: This section will use only TCN, combined with 1D-CNN and GCN (CGNN), combined with GAT and TCN (GTNN), combined with GraphSAGE-TCN (GSTNN), multivariate time series graph neural network (MTGNN) and EEFL for comparison to verify the effectiveness of the proposed method in fault diagnosis. Table 2 and Fig. 6 show the comparison results.

**Table 2:** Comparison results of fault diagnosis.

Algorithm	Accuracy	Precision	Recall	${F_1}$score
TCN	89.40%±0.55%	88.80%±0.61%	88.50%±0.64%	88.60%±0.58%
CGNN	91.00%±0.42%	90.50%±0.48%	90.20%±0.51%	90.30%±0.45%
GTNN	92.30%±0.36%	91.80%±0.40%	91.50%±0.43%	91.60%±0.34%
GSTNN	93.50%±0.31%	93.00%±0.35%	92.80%±0.38%	92.90%±0.30%
MTGNN	94.75%±0.28%	94.40%±0.32%	94.20%±0.34%	94.30%±0.26%
EEFL	95.80%±0.22%	95.50%±0.25%	95.20%±0.28%	95.30%±0.20%

In the fault diagnosis task, the EEFL hybrid diagnosis model designed in this paper demonstrated optimal performance in identifying multiple fault types. Experimental results show that TCN, which relies solely on time series modeling, can effectively capture the dynamic characteristics of link operation indicators. However, due to its lack of modeling of topological dependencies, its accuracy is only 89.40%, which leaves some limitations in identifying data contamination faults. CGNN, which extracts time series features through convolution and combines it with graph convolution, achieves improved performance, reaching an accuracy of 91.00%, but still suffers from information loss in complex dependency scenarios. GTNN leverages an attention mechanism to enhance its ability to identify key nodes and edges, further increasing its accuracy to 92.30%. GSTNN exhibits advantages in sample expansion and high-order feature modeling, improving its accuracy to 93.50%. In contrast, the EEFL fusion model proposed in this paper takes into account both timing dependency and topological dynamic characteristics, effectively solving the problem of insufficient discrimination of complex fault modes by a single method. It outperforms the comparison method in all indicators, ultimately achieving 95.80% accuracy, 95.50% recall, 95.20% precision, and 95.30% ${F_1}$score, fully verifying its accuracy and robustness in multi-type link fault diagnosis.

(3) Verification experiment of dynamic threshold alarm: In order to verify the effectiveness of the dynamic threshold in the EEFL method proposed in this paper, this section compares it with the static threshold (ST), moving average threshold (MAT), quantile threshold (QT), exponentially weighted moving threshold (EWMT) and unsupervised multivariate time series anomaly detection method based on stochastic recurrent neural networks (OmniAnomaly) algorithm.

**Table 3:** Comparison results of dynamic threshold alarm.

Algorithm	Accuracy	Precision	Recall	${F_1}$score	FAR	MAR	ALT(minutes)
ST	84.20%	83.50%	83.00%	83.20%	16.5%	17.0%	1.2
MAT	86.50%	86.00%	85.50%	85.70%	14.0%	14.5%	2.5
QT	87.80%	87.30%	87.00%	87.10%	12.7%	13.0%	3.1
EWMT	90.20%	89.70%	89.40%	89.50%	10.3%	10.6%	4.6
OmniAnomaly	92.40%	91.90%	91.70%	91.80%	8.1%	8.3%	5.8
EEFL	93.80%	93.40%	93.10%	93.20%	6.6%	6.9%	8.2

**Fig. 7:**
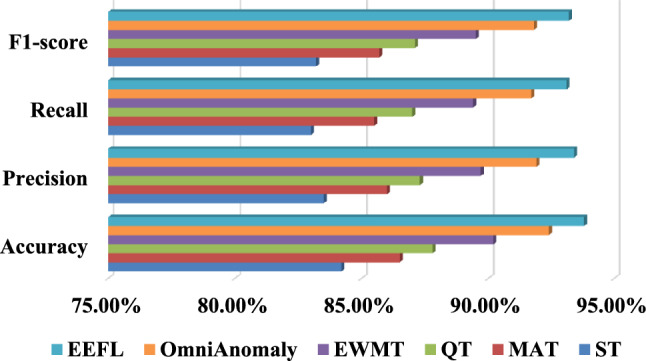
Comparison results of dynamic threshold alarm.

Table 3 shows the accuracy, precision, recall, ${F_1}$score, false alarm rate (FAR), missing alarm rate (MAR) and alarm lead time (ALT) of different algorithms. Fig. 7 shows the comparison results of accuracy, precision, recall, ${F_1}$score. Specifically, the traditional ST method relies solely on fixed rules to trigger alerts. While simple to implement, it lacks adaptability to changes in data distribution, resulting in the worst performance (84.20% accuracy). The MAT method can mitigate the impact of data drift to some extent, improving accuracy to 86.50%, but still suffers from high false alarm rates in sudden anomaly scenarios. The QT method outperforms the previous two in adaptability, but lacks global optimization capabilities, achieving an accuracy of 87.80%. EWMA significantly reduces false positives (improved precision) and misses in emergencies (improved recall) by rapidly adjusting the threshold when a new distribution emerges. In contrast, the Bayesian optimization plus online learning (EEFL) dynamic threshold mechanism proposed in this paper can quickly adapt to changes in data distribution while ensuring global optimality. Ultimately, it significantly outperforms other methods in accuracy (93.8%), recall (93.40%), precision (93.10%), and ${F_1}$ score (93.20%), demonstrating that this mechanism has strong practical value and robustness in full-link alarm tasks.

(4) Ablation study: To verify the necessity of each module, this section compiles ablation experiments that implement different functions. Specifically, we removed the dynamic edge weighting module (GCNS), the TCN temporal encoder (CGNN), the GNN topological encoder (TCN), and the dynamic threshold mechanism (ST) in turn to evaluate their individual impacts. The results shown in Table 4 confirm that each component makes a nontrivial contribution to the overall performance. Notably, removing either the TCN or GNN significantly decreases both accuracy and ${F_1}$score, demonstrating that temporal and topological modeling are complementary. The proposed dynamic threshold further improves early warning precision and stability.

**Table 4:** Results of ablation study.

Algorithm	Accuracy	Precision	Recall	${F_1}$score
GCNS	91.20%	90.50%	90.00%	90.20%
EEFL-pattern recognition	97.20%	96.80%	96.50%	96.60%
TCN	89.40%	88.80%	88.50%	88.60%
CGNN	91.00%	90.50%	90.20%	90.30%
EEFL-fault diagnosis	93.80%	93.40%	93.10%	93.20%
ST	84.20%	83.50%	83.00%	83.20%
EEFL-Early Warning	93.80%	93.40%	93.10%	93.20%

(5) Comparison of runtime: All runtime performance evaluations were conducted on a server equipped with an Intel Xeon Gold 6248R CPU (2.40GHz), 256GB of RAM, and a single NVIDIA A100 GPU with 40GB of VRAM. Models were implemented in PyTorch. For inference latency measurements, a consistent batch size of 64 was used. The results of training time and inference latency are shown in Table 5. In terms of training time, a clear correlation with model complexity is observed. The SOTA models, TGN and MTGNN, required the longest training durations (4.8 and 4.1 hours, respectively) due to their sophisticated architectures involving memory updates or computationally expensive graph learning layers. In contrast, our EEFL modules for pattern recognition (3.5 hours) and fault diagnosis (3.8 hours) demonstrate a significant efficiency advantage over these SOTA counterparts while being more powerful than faster baselines like GCNS (1.2 hours). This favorable balance stems from our framework’s targeted design, which efficiently utilizes the pre-defined data lineage graph, thereby avoiding the overhead of learning the graph structure from scratch. Regarding inference latency, which is critical for real-time applications, our EEFL framework proves highly practical. For instance, the EEFL fault diagnosis module’s latency of 20.5 ms per batch is competitive with MTGNN (22.8 ms), confirming its suitability for online deployment. Notably, the EEFL early warning module, with its near-instantaneous online update latency of 1.2 ms, is substantially more efficient for real-time adaptation than the deep learning-based OmniAnomaly (19.6 ms), showcasing its superior design for dynamic operational environments.

**Table 5:** Comparison results of runtime.

Task	Algorithm	Training Time (hours)	Inference Latency ( ms/batch )	Task	Algorithm	Training Time (hours)	Inference Latency ( ms/batch )
Link Pattern Recognition	GCNS	1.2	8.5	Fault Diagnosis	TCN	1.5	9.2
	GraphSAGE	1.8	12.1		CGNN	2.2	15.7
	GAT	2.5	16.3		MTGNN	4.1	22.8
	WGCN	2.1	14.8		EEFL-Diagnosis	3.8	20.5
	TGN	4.8	25.4	Threshold Alarm	OmniAnomaly	3.2	19.6
	EEFL-Pattern	3.5	18.2		EEFL-Warning	0.5 (Offline)	1.2 (Online)

### Distribution network simulation evaluation

This section uses the IEEE-33 and IEEE-123 test systems as the framework for network topology and power flow simulation. MBased on MATPOWER simulations, including 33/123 buses and 32/122 lines. Node features include voltage magnitude, phase angle, and active/reactive injections; edge features include real/reactive line flow and line loading ratio. Fault labels (line outage, load surge, bad data) were generated through controlled simulations. Specific verification experimental results are shown below.

**Table 6:** Comparison results of link pattern recognition of various algorithms.

Algorithm	Accuracy	Precision	Recall	${F_1}$score
GCNS	79.01%±0.65%	79.43%±0.71%	78.60%±0.75%	79.01%±0.68%
GraphSAGE	80.34%±0.58%	80.61%±0.62%	80.12%±0.65%	80.36%±0.55%
GAT	81.67%±0.51%	81.95%±0.55%	81.40%±0.59%	81.67%±0.49%
WGCN	82.98%±0.45%	83.29%±0.49%	82.81%±0.52%	83.05%±0.43%
TGN	83.85%± 0.41%	84.10%±0.44%	83.60%±0.48%	83.85%± 0.39%
EEFL	84.76%±0.35%	85.09%±0.38%	84.60%±0.41%	84.84%± 0.32%

**Fig. 8:**
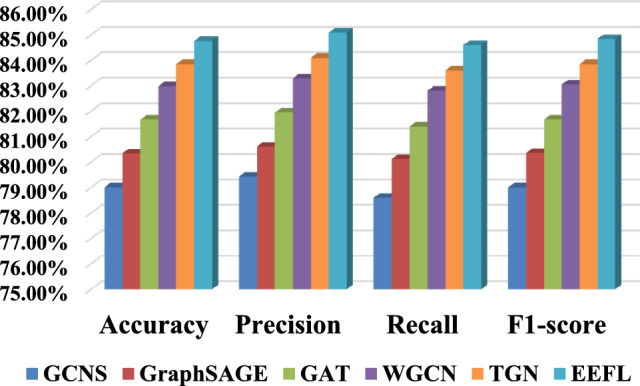
Comparison results of link pattern recognition of various algorithms.

1. Verification experiment of link pattern recognition: The results are shown in Table 6 and Fig. 8. Experimental results show that the EEFL method achieves the best performance on the link pattern recognition task, achieving the best results across all evaluation metrics (Accuracy = 84.76%, Precision = 85.09%, Recall = 84.60%, ${F_1}$score = 84.84%). Compared to the basic GCN, the EEFL model improves accuracy and ${F_1}$score by 5.75% and 5.83%, respectively, demonstrating that the introduction of edge temporal feature encoding and edge weight normalization effectively enhances the model’s ability to discriminate link patterns. Further comparisons show that GraphSAGE and GAT also show some improvement over GCN, indicating that diverse aggregation methods that capture neighbor features in this task can improve the generalization of topological pattern recognition. Overall, this experiment demonstrates that the fusion of temporal edge features and topological information can significantly improve the accuracy and robustness of data link pattern recognition.

**Table 7:** Comparison results of fault diagnosis.

Algorithm	Accuracy	Precision	Recall	${F_1}$score
TCN	87.23%±0.62%	87.35%±0.68%	87.02%±0.71%	87.18%±0.65%
CGNN	89.21%±0.53%	89.36%±0.59%	89.02%±0.62%	89.19%±0.51%
GTNN	90.58%±0.46%	90.69%±0.50%	90.40%±0.54%	90.54%±0.44%
GSTNN	91.80%±0.40%	91.89%±0.44%	91.72%±0.47%	91.80%±0.38%
MTGNN	92.65%±0.36%	92.70%±0.39%	92.50%±0.42%	92.60%±0.34%
EEFL	93.52%±0.29%	93.61%±0.31%	93.40%±0.35%	93.50%±0.27%
2. Verification experiment of fault diagnosis: In the fault diagnosis task, experimental results (as shown in the Table 7 and Fig. 9) show that the EEFL achieved the highest precision, recall, and F1 score, significantly outperforming other control models. Compared to the TCN alone, the EEFL model improved accuracy by approximately 6.3% and ${F_1}$ score by approximately 7.2%, demonstrating that relying solely on temporal features is insufficient for capturing complex link faults and requires joint modeling incorporating graph structural information. While methods such as CGNN and GTNN also achieved comparable results, their overall performance remained below that of the EEFL. This suggests that in this scenario, the causal convolution-based TCN can better model long-term dependencies, and combining topological features can further enhance the diagnostic capabilities for complex multi-class faults. These experimental results demonstrate the robustness and generalization capabilities of the EEFL model for complex distribution network fault diagnosis.

**Fig. 9:**
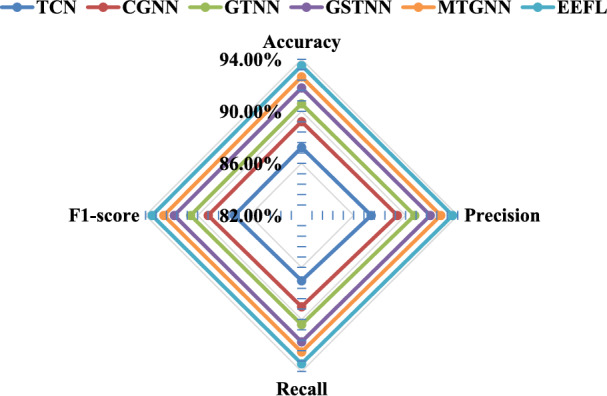
Comparison results of fault diagnosis.

**Fig. 10:**
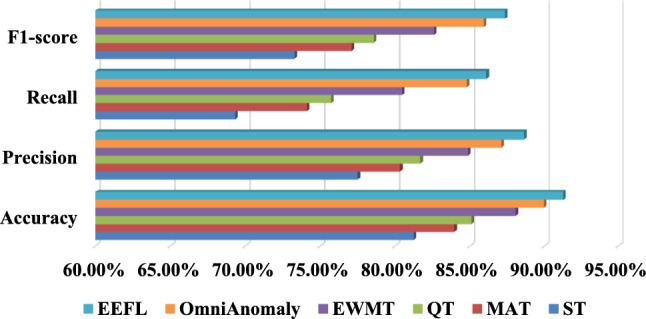
Comparison results of dynamic threshold alarm.

**Table 8:** Comparison results of dynamic threshold alarm.

Algorithm	Accuracy	Precision	Recall	${F_1}$score	FAR	MAR	ALT(simulation steps)
ST	81.24%	77.50%	69.32%	73.28%	22.5%	30.7%	0.8
MAT	83.95%	80.32%	74.10%	77.10%	19.7%	25.9%	1.5
QT	85.10%	81.68%	75.73%	78.57%	18.3%	24.3%	1.9
EWMT	88.04%	84.87%	80.45%	82.60%	15.1%	19.5%	2.8
OmniAnomaly	89.95%	87.10%	84.80%	85.93%	12.9%	15.2%	4.1
EEFL	91.27%	88.61%	86.10%	87.34%	11.4%	13.9%	5.3
3. Verification experiment of dynamic threshold alarm: In the threshold warning task, different threshold selection methods showed significant differences in their effectiveness in suppressing false alarms and missed alarms (as shown in the Table 8 and Fig. 10). Experimental results showed that the traditional fixed threshold performed the worst, suffering from high missed alarms and long alert delays. The introduction of the MAT and QT strategies improved overall performance, reaching F1 scores of 77.10% and 78.57%, respectively. EWMA further improved performance. Ultimately, the EEFL method (Bayes+FTRL dynamic threshold) achieved the best performance, outperforming the comparison methods across all evaluation metrics. This demonstrates that the threshold adaptation mechanism based on Bayesian optimization and online learning can effectively track changes in data distribution, balancing precision and recall, and possesses practical application value.
4. Ablation study: The ablation experimental model GCNS, CGNN, TCN and ST are used to demonstrate the necessity of each module. As sketched in Table 9 the basic GCNS, utilizing only static topology for inference, exhibits the lowest performance. However, the addition of an edge-time-aware module significantly improves pattern recognition capabilities, indicating that edge-level temporal information plays a crucial role in distinguishing link structure patterns. Furthermore, while using only TCN or GNN can improve performance, it remains significantly lower than fault diagnosis that integrates spatial topology and temporal dependencies. This verifies the complementarity of temporal dependencies and link topology, demonstrating that using either feature alone is insufficient to achieve optimal performance. In addition, ST performs significantly poorly in early warning tasks, but the introduction of Bayesian optimization and online learning significantly improves the quality of early warning decisions. Overall, these results fully demonstrate that the three modules of EEFL each bring stable and significant independent gains, while complete integration can further create a synergistic effect, resulting in optimal diagnostic and early warning performance.

**Table 9:** Results of ablation study.

Algorithm	Accuracy	Precision	Recall	${F_1}$score
GCNS	79.01%	79.43%	78.60%	79.01%
EEFL-pattern recognition	84.76%	85.09%	84.60%	84.84%
TCN	87.23%	87.35%	87.02%	87.18%
CGNN	89.21%	89.36%	89.02%	89.19%
EEFL-fault diagnosis	93.52%	93.61%	93.40%	93.50%
ST	81.24%	77.50%	69.32%	73.28%
EEFL-Early warning	91.27%	88.61%	86.10%	87.34%

**Table 10:** Comparison results of runtime.

Task	Algorithm	Training time (hours)	Inference latency (ms/batch)	Task	Algorithm	Training time (hours)	Inference latency (ms/batch)
Link pattern recognition	GCNS	0.9	1.5	Fault diagnosis	TCN	1.1	1.8
	GraphSAGE	1.3	2.1		CGNN	1.7	2.5
	GAT	1.9	2.8		MTGNN	3.2	3.8
	WGCN	1.6	2.6		EEFL-Diagnosis	2.9	3.5
	TGN	3.5	4.1	Threshold alarm	OmniAnomaly	2.5	3.1
	EEFL-Pattern	2.6	3.2		EEFL-Warning	0.4 (Offline)	1.1 (Online)
5. Comparison of runtime: Table 10 presents the runtime performance on the distribution network simulation dataset, which is characterized by a very large number of small-scale graphs. The analysis provides insights into the models’ scalability in high-volume scenarios. For training time, despite the small graph size, the sheer volume of samples (576,000) resulted in substantial training durations across all models. The relative performance ranking, however, remained consistent with the enterprise dataset. Our EEFL modules (2.6 hours for pattern recognition and 2.9 hours for fault diagnosis) were once again more efficient than the SOTA models TGN (3.5 hours) and MTGNN (3.2 hours), underscoring the computational benefits of our streamlined architecture. In terms of inference latency, all models exhibited significantly lower latencies due to the small graph size (e.g.,  123 nodes), making them well-suited for high-throughput processing. For example, the EEFL fault diagnosis module’s latency was only 3.5 ms per batch. Even in this low-latency regime, our EEFL framework maintained a competitive speed relative to the SOTA models. This demonstrates that our proposed framework is not only effective for large, monolithic graphs but is also highly scalable and efficient for scenarios involving the rapid, sequential processing of smaller graph structures. The EEFL early warning mechanism, with an online update time of 1.1 ms, again proves its exceptional efficiency for practical deployment.

## Conclusion

We propose an end-to-end, full-link intelligent analysis method based on data lineage to address the intelligent operation and maintenance requirements for complex links from the data center to the application scenario end. By introducing dynamic graph lineage modeling and GNN, this paper achieves high-precision recognition of link topology patterns. The TCN-GNN hybrid diagnostic model designed further effectively integrates temporal dependencies and topological structure characteristics, enabling accurate classification of multiple types of link faults. Furthermore, the dynamic threshold warning mechanism combining Bayesian optimization and FTRL significantly reduces false positive and false negative rates, enhancing the robustness of the system in dynamic environments. Experimental results demonstrate that the proposed method outperforms existing methods in link pattern recognition and fault prediction accuracy, recall rate, and ${F_1}$ score, validating its effectiveness and practical value. Future work will further consider scalability research in cross-domain heterogeneous data environments, as well as engineering deployment and application in real-time operation and maintenance systems.

## Data Availability

The datasets analysed during the current study are available from the corresponding author on reasonable request.

## Abbreviations

EEFL: End-to-end full-link intelligent analysis framework
GNNs: Graph neural networks
TCN: Temporal convolutional network
FTRL: Follow-the-regularized-leader
GCN: Graph convolution neural networks
GP: Gaussian process
GraphSAGE: Graph sample and aggregate
GAT: Graph attention network
WGCN: Weighted graph convolution neural networks
CDNN: Combined with 1D-CNN and GCN
CTNN: Combined with GAT and TCN
GSTNN: Combined with GraphSAGE-TCN
TGN: Temporal graph network
MTGNN: Multivariate time series graph neural network
ST: Static threshold
MAT: Moving average threshold
QT: Quantile threshold
EWMT: Exponentially weighted moving threshold
FAR: False alarm rate
MAR: Missing alarm rate
ALT: Alarm lead time
